# The Upregulation of AIM2 in the Central Nucleus of the Amygdala Correlates with Pain Induced by Tooth Movement

**DOI:** 10.3390/ijms27104647

**Published:** 2026-05-21

**Authors:** Rui Wang, Yutong Guo, Weining Wang, Yuhuan Jiang, Tingting Lin, Wenhui Liang, Bing Qi, Hu Qiao

**Affiliations:** Key Laboratory of Shaanxi Province for Craniofacial Precision Medicine Research, College of Stomatology, Xi’an Jiaotong University, Xi’an 710004, China; wr1215707121@163.com (R.W.); 15803597151@163.com (Y.G.); 15529881830@163.com (W.W.); huaner0919@163.com (Y.J.); mmltt75@163.com (T.L.); orthodontist_liang@163.com (W.L.); q8325179406@163.com (B.Q.)

**Keywords:** tooth movement-induced pain, the central nucleus of the amygdala, AIM2, behavior assessment

## Abstract

Pain is an unavoidable experience during orthodontic treatment. The central nucleus of the amygdala (CeA) plays a key role in regulating emotion and pain. Meanwhile, Absent in Melanoma 2 (AIM2) has been demonstrated in multiple neuroinflammatory and pain models for promoting inflammatory responses then enhancing nociceptive signaling. However, its role in pain caused by orthodontic tooth movement has not yet been clarified. In this study, C57BL/6J mice were used to establish an experimental tooth movement (ETM) model and were assigned to a control group, sham group, and experimental group. The face grooming and von Frey results showed that pain behaviors reached a peak on 1 d and returned to baseline levels by 7 d. After 14 days of continuous force application, mice developed obvious anxious behaviors and progressively worsened over time. The Western blot results revealed that tooth movement significantly increased AIM2 protein expression in the CeA. This was accompanied by a marked upregulation of NLRP3, caspase-1 and pp65. These findings suggest a potential role of NLRP3-NF-κB signaling in orthodontic tooth movement and also provide a new central target for the precise regulation of orthodontic pain.

## 1. Introduction

Malocclusion is a highly prevalent oral health condition. According to the World Health Organization, it ranks as the third most common oral health issue globally, following only dental caries and periodontal disease [1]. The majority of patients with malocclusion require orthodontic treatment. Research indicates that pain occurs in 72–100% of orthodontic treatment cases [2]. This significantly impacts patients’ treatment experience and increases anxiety. Such anxiety reduces compliance and prolongs treatment duration, ultimately creating a vicious cycle. Existing research suggests a mutually reinforcing relationship between pain and anxiety. Pain can elevate patients’ anxiety levels, while anxiety states can amplify pain perception through central pain modulation mechanisms [3]. This further reduces treatment compliance, prolongs orthodontic treatment cycles, and creates a vicious cycle. Therefore, effectively managing orthodontic treatment-related pain and associated emotional factors is crucial for improving the patient experience and treatment efficacy.

The amygdala is a core brain structure involved in emotional regulation and stress modulation. The central nucleus of the amygdala (CeA) serves as the primary output nucleus of the amygdala. It connects to the brainstem and hypothalamus while interacting with multiple regions, including the cerebral cortex and hippocampus. It regulates physiological responses to emotions and pain. Studies indicate that the surgical destruction of the CeA alleviates pain in rats, further confirming its crucial role in pain processing [4]. Previous studies have explored the classic pain molecule TRP family [5], revealing that these ion channels play a crucial role in sensory transmission during orthodontic pain. However, targeting TRP channels alone does not fully alleviate tooth movement pain [6]. This suggests that pain generation involves more than peripheral receptor pathways, potentially relying on complex central regulatory mechanisms. Recent studies indicate that the CeA in the central nervous system not only participates in processing acute pain signals but also plays a crucial role in the formation and maintenance of chronic pain [7]. However, the precise mechanisms underlying the CeA’s role in orthodontic pain remain unclear. Further research is needed to elucidate how distinct neural circuits and molecular signaling pathways within the CeA influence pain sensitivity and its affective dimensions.

Absent in Melanoma 2 (AIM2) is a cytoplasmic pattern recognition receptor that plays a central role in inflammatory responses [8]. Research demonstrates that AIM2 not only functions in peripheral immunity but also participates in inflammatory responses within the central nervous system. It promotes caspase-1 activation by recognizing double-stranded DNA in the cytoplasm to activate inflammasomes [9]. Research indicates that AIM2 is involved in both infection-induced systemic inflammation and various aseptic inflammations [10]. Relevant examples include neuropathic pain, chronic pain, and atherosclerosis. Orthodontic tooth movement-induced pain is often accompanied by complex neuroinflammatory responses. Within the central nervous system, the activation of AIM2 inflammasomes may be a key trigger in this process. However, the mechanism by which AIM2 contributes to orthodontic tooth movement pain and its association with the CeA remain unclear and warrant further investigation.

NOD-like receptor family pyrin domain-containing 3 (NLRP3) is a crucial inflammasome sensor that recognizes intracellular pathological signals and activates immune responses within cells. In inflammatory pain responses, NLRP3 activates downstream caspase-1, thereby initiating the maturation and release of inflammatory mediators [11]. Studies indicate that NF-κB is a key factor essential for NLRP3 activation [12], a process that can lead to the sustained elevation of neuronal excitability, thereby inducing chronic pain and anxiety. In orthodontic pain models, the phosphorylation of pp65 within the CeA brain region and its mediated upregulation of AIM2 expression may serve as the initiating step triggering subsequent caspase-1 activation and anxiety-like behaviors. However, the precise relationship between AIM2 and NLRP3 within the CeA remains unclear. Therefore, this study aims to investigate the expression changes in these two molecules in the CeA and their roles in orthodontic pain to uncover novel pathways for alleviating orthodontic pain.

## 2. Results

### 2.1. Micro-CT and Rate of Tooth Movement

**Figure 1:**
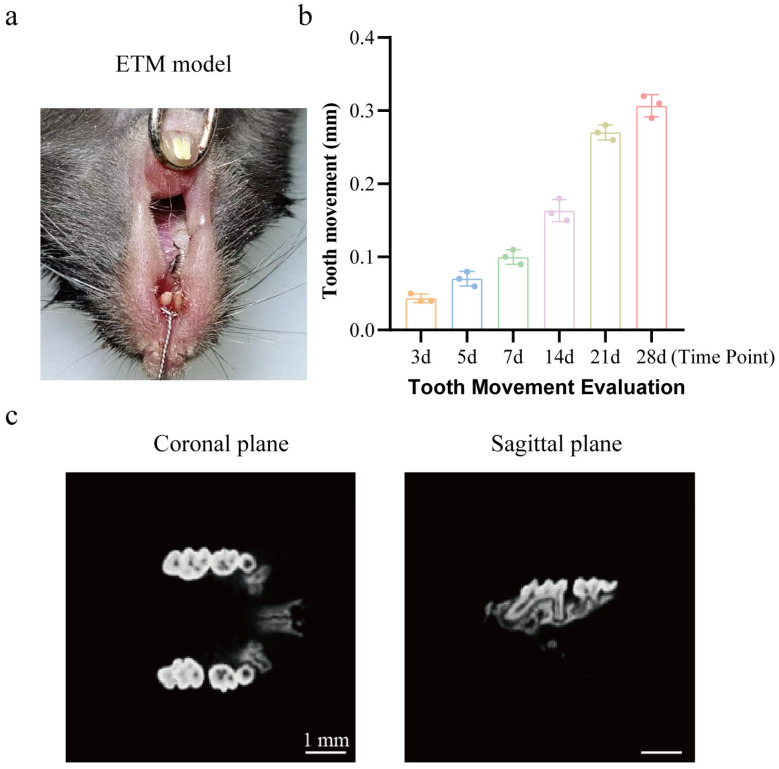
Micro-CT performance of ETM. (**a**) ETM model diagram. (**b**) ETM distance from 3 d to 28 d (mm). (**c**) Micro-CT image. Statistical analysis was performed using one-way repeated measures (RM) ANOVA. Data are expressed as means ± standard error of mean (SEM). Abbreviation: ETM, experimental tooth movement.

The results are shown in Figure 1. The micro-CT results reveal that the upper right first molar in the EG (experimental group) exhibited significant mesial movement, accompanied by the widening of the periodontal ligament space, indicating successful orthodontic modeling. Under the sustained application of orthodontic force, the maxillary right first molar exhibited significant mesial movement, with displacement increasing over time (one-way repeated measures (RM) ANOVA, *F*(1.786, 3.573) = 413.2, *p* < 0.0001; *n* = 3 per time point). Tooth movement progressed slowly from 3 d to 7 d (3 d vs. 7 d, *p* = 0.2987), accelerated markedly starting at 14 d (7 d vs. 14 d, *p* = 0.0406), and reached a peak at 28 d (14 d vs. 28 d, *p* = 0.0081).

### 2.2. Face Grooming Behavior After ETM

**Figure 2:**
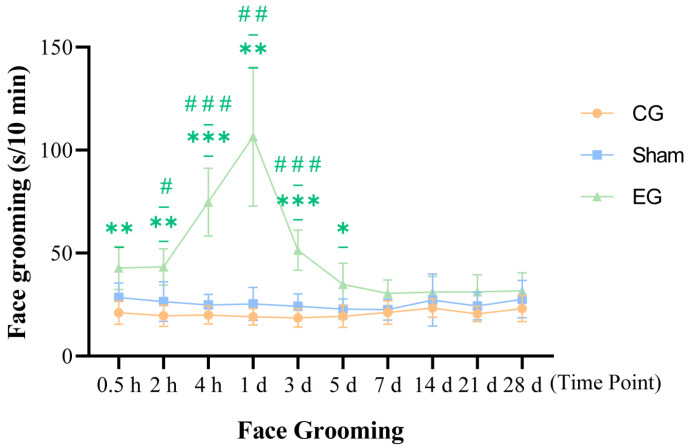
Face grooming behavior after ETM. *n* = 6, * *p* < 0.05, ** *p* < 0.01, *** *p* < 0.001, compared with control group; # *p* < 0.05, ## *p* < 0.01, ### *p* < 0.001, compared with sham group. Statistical analysis was performed using 3 × 10 mixed two-way ANOVA, followed by one-way ANOVA and Tukey’s post hoc test for group comparisons at each time point. Data are expressed as means ± standard error of mean (SEM). Blue label: sham group. Green label: experimental group. Orange label: control group. Abbreviations: ETM, experimental tooth movement; CG, control group; EG, experimental group.

Face grooming activity in mice was observed at 0.5 h, 2 h, 4 h, 1 d, 3 d, 5 d, 7 d, 14 d, 21 d, and 28 d (Figure 2). A 3 × 10 mixed two-way ANOVA revealed significant main effects for both Group (*F*(2, 15) = 113.0, *p* < 0.0001) and Time (*F*(3.561, 53.42) = 12.26, *p* < 0.0001), along with a significant interaction between the two factors (*F*(18, 135) = 13.54, *p* < 0.0001; *n* = 6 per group). Both the EG and sham group exhibited grooming behavior, but the EG demonstrated significantly higher activity compared to the CG and sham group starting from 0.5 h. In the EG, face grooming behavior increased and reached a peak at 1 d, significantly exceeding that in the CG (*p* = 0.0031) and sham group (*p* = 0.0037) at this time point. Following this peak, grooming activity gradually declined. By day 7, no significant differences were observed between the EG and sham group (*p* = 0.0975), indicating a gradual alleviation of the orthodontic force-induced pain.

### 2.3. Von Frey Mechanical Threshold Behavior After ETM

**Figure 3:**
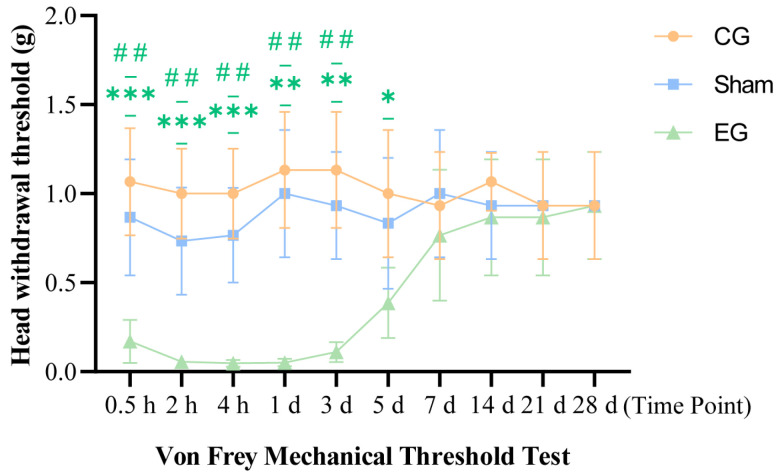
Von Frey mechanical threshold behavior after ETM. *n* = 6, * *p* < 0.05, ** *p* < 0.01, *** *p* < 0.001, compared with control group; ## *p* < 0.01, compared with sham group. Statistical analysis was performed using 3 × 10 mixed two-way ANOVA, followed by one-way ANOVA and Tukey’s post hoc test for comparisons among CG, sham group, and EG at each time point. Data are expressed as means ± standard error of mean (SEM). Blue label: sham group. Green label: experimental group. Orange label: control group.

The von Frey test results are shown in Figure 3. A 3 × 10 mixed two-way ANOVA revealed significant main effects for Group (*F*(2, 15) = 44.61, *p* < 0.0001), Time (*F*(5.939, 89.09) = 4.472, *p* = 0.0006), and their interaction (*F*(18, 135) = 4.487, *p* < 0.0001; *n* = 6 per group). The head withdrawal threshold in the experimental group began to decrease after force application at 0.5 h (EG vs. CG, *p* = 0.0009) and remained at extremely low levels between 2 h and 1 d (at 1 d: EG vs. CG, *p* = 0.0011; EG vs. sham, *p* = 0.0030). Subsequently, it gradually increased starting from 3 d and largely recovered to baseline levels by 7 d (at 7 d: EG vs. sham, *p* = 0.5268). No significant differences were observed among the three groups between 14 d and 28 d (all *p* > 0.05). These results indicate that the pain response in the experimental group was primarily concentrated within the first 3 d following force application.

### 2.4. Open Field Test (OFT) Behavior After ETM

**Figure 4:**
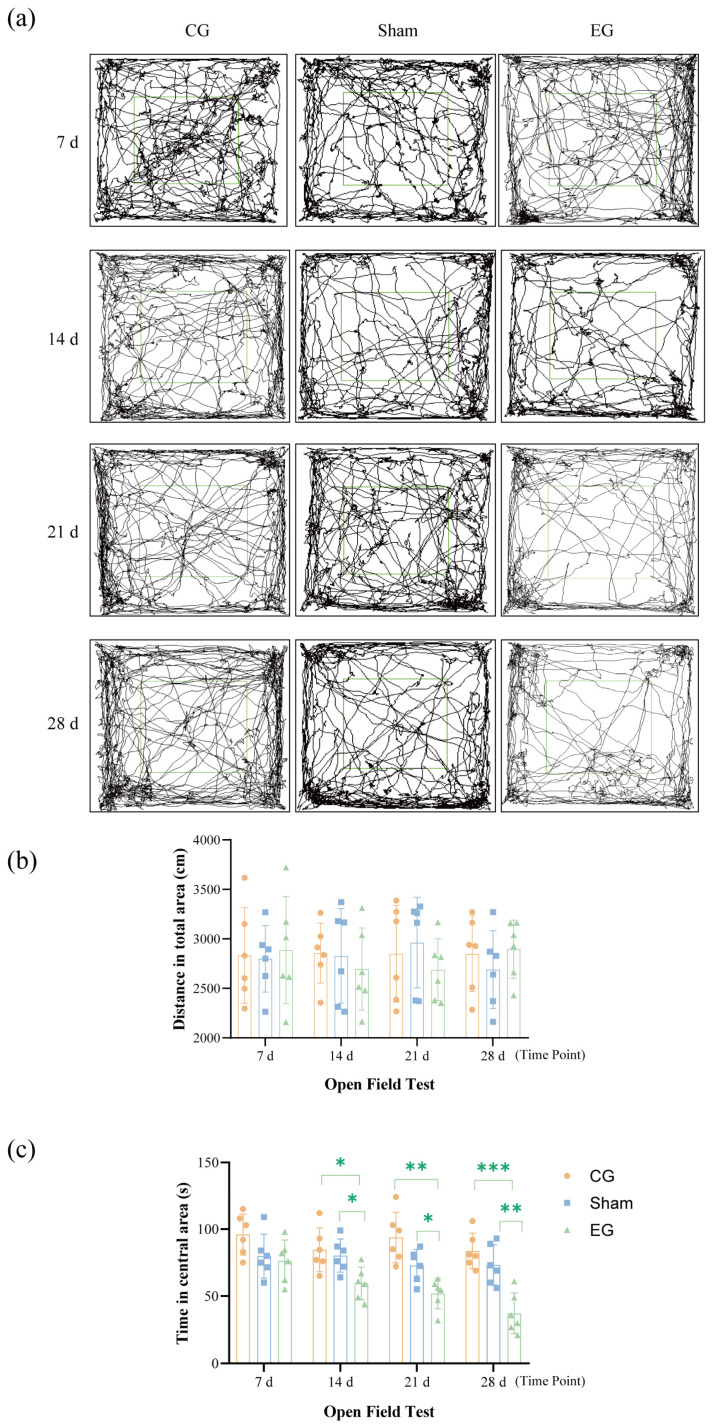
OFT behavior after ETM. (**a**) OFT trajectory diagram. (**b**) Results of distance in total area. (**c**) Results of time in central area. *n* = 6, * *p* < 0.05, ** *p* < 0.01, *** *p* < 0.001. Statistical analysis was performed using 3 × 4 mixed two-way ANOVA, followed by one-way ANOVA at each specific time point for group comparisons. Data are expressed as means ± standard error of mean (SEM).

Compared with the control group and sham group, the experimental group exhibited a significant reduction in total locomotor distance, a markedly shorter dwell time in the central area (Figure 4b), and a significantly lower frequency of entering the central area (Figure 4c). A 3 × 4 mixed two-way ANOVA for central dwell time revealed significant main effects for Group (*F*(2, 15) = 55.34, *p* < 0.0001) and Time (*F*(2.494, 37.41) = 4.584, *p* = 0.0113; *n* = 6 per group). This trend gradually worsened starting from 14 d (EG vs. CG, *p* = 0.0348; EG vs. sham, *p* = 0.0361) and reached its most pronounced level by 28 d (EG vs. CG, *p* = 0.0006; EG vs. sham, *p* = 0.0051), indicating that the mice displayed pronounced anxiety-like behavioral manifestations. These findings suggest the potential emergence of pronounced anxiety-like manifestations in mice.

### 2.5. Elevated Plus Maze (EPM) Behavior After ETM

**Figure 5:**
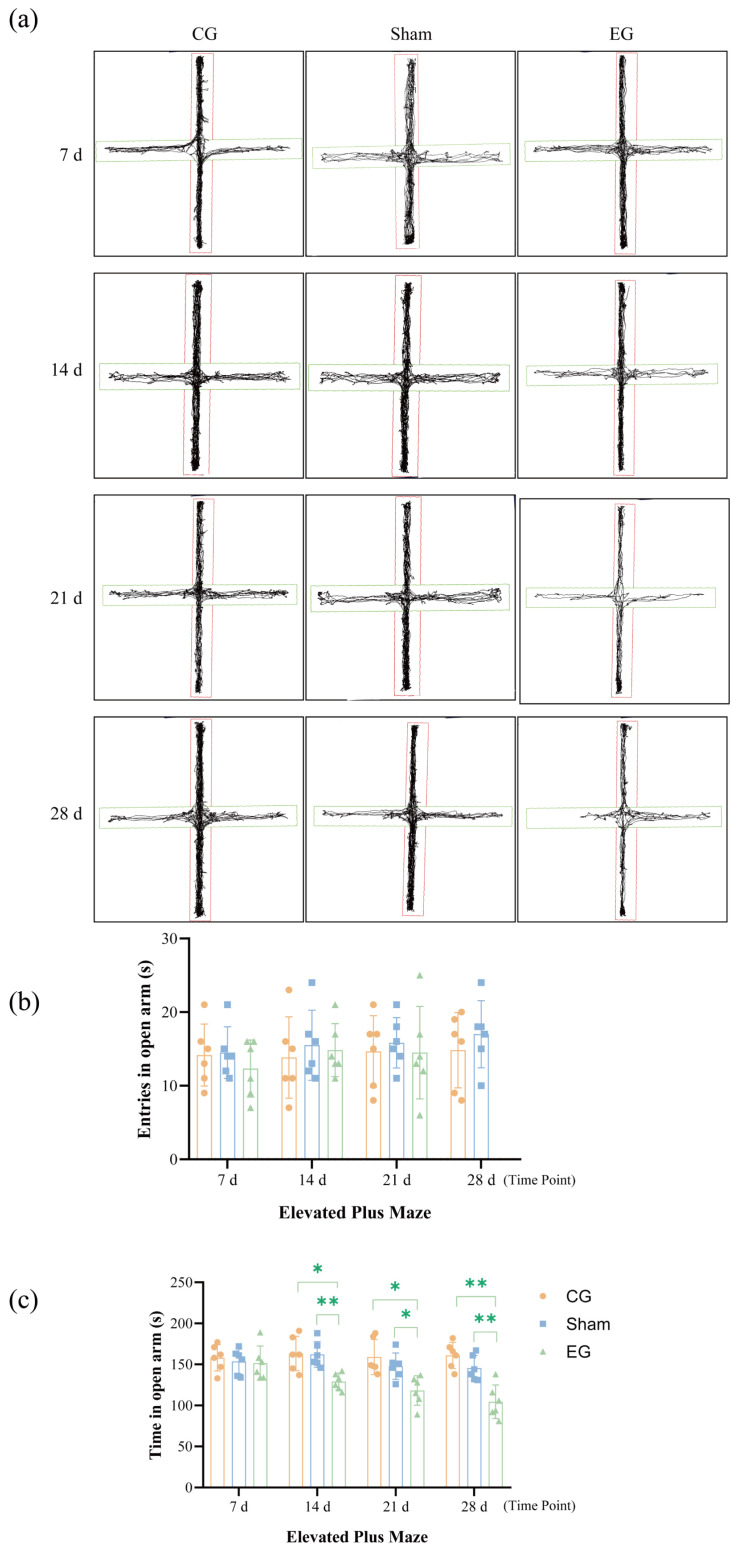
EPM behavior after ETM. (**a**) EPM trajectory diagram. Red: closed arms; Green: open arms. (**b**) Results of entries in open arm. (**c**) Results of time in open arm. *n* = 6, * *p* < 0.05, ** *p* < 0.01. Statistical analysis was performed using 3 × 4 mixed two-way ANOVA, followed by one-way ANOVA at each specific time point for group comparisons. Data are expressed as means ± standard error of mean (SEM).

The results are shown in Figure 5. The EG exhibited reduced total locomotor activity within the maze compared to the other two groups. A 3 × 4 mixed two-way RM ANOVA conducted on the duration of stay in the open arms (Figure 5b) revealed significant main effects for Group (*F*(2, 15) = 16.63, *p* = 0.0002) and Time (*F*(2.860, 42.90) = 4.849, *p* = 0.0061), as well as a significant interaction (*F*(6, 45) = 3.069, *p* = 0.0133; *n* = 6 per group). As the exposure time increased, both the duration of stay and the number of entries into the open arms significantly declined. Tukey’s post hoc tests revealed that this anxiety-like pattern began to emerge around 14 d (EG vs. CG, *p* = 0.0199; EG vs. sham, *p* = 0.0055) and gradually intensified until it peaked at 28 d (EG vs. CG, *p* = 0.0010; EG vs. sham, *p* = 0.0080). These observations suggest that sustained orthodontic mechanical stimulation may induce pronounced and time-dependent anxious behavioral responses in mice.

### 2.6. Upregulation of AIM2 Expression in Central Amygdala

**Figure 6:**
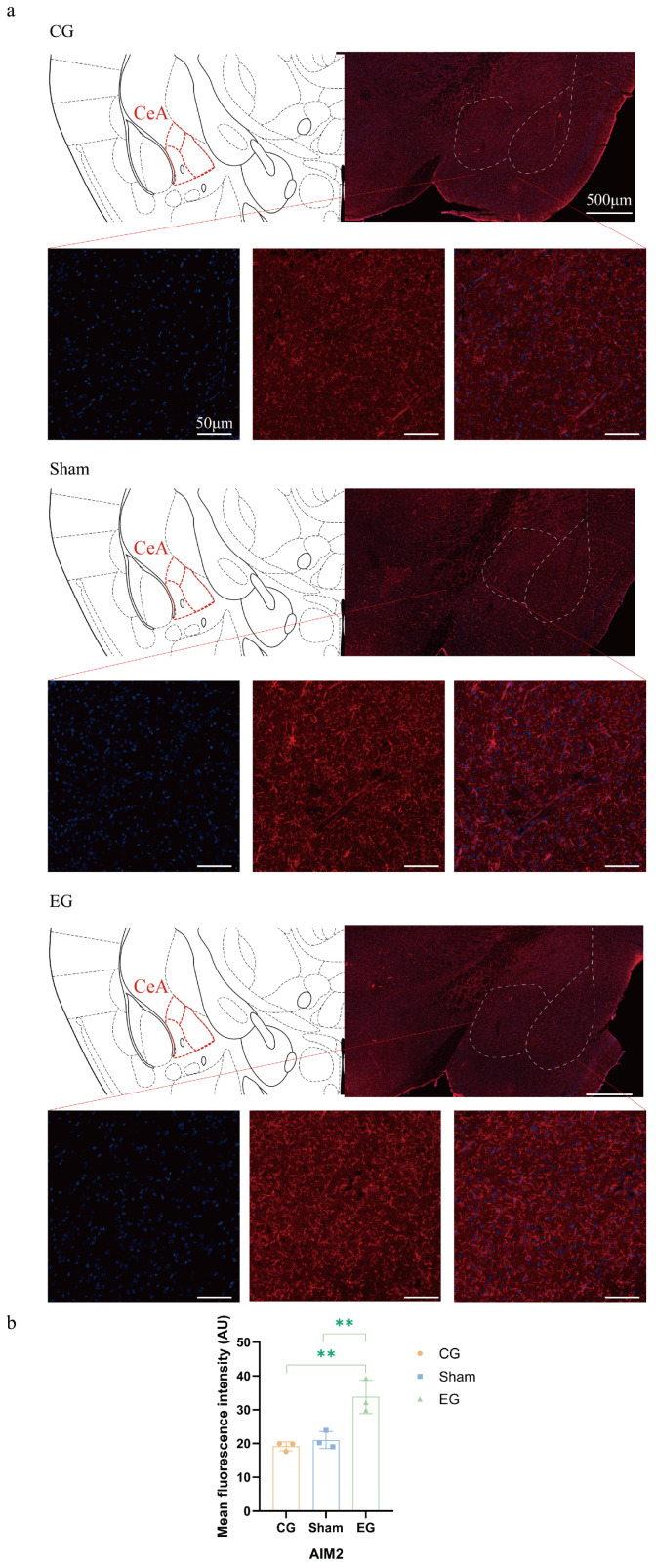
Immunofluorescence staining of AIM2 in CeA at 1 d. (**a**) Representative immunofluorescence images showing AIM2 expression in CeA of CG, sham, and EG. Blue indicates DAPI staining, and red indicates AIM2 staining. (**b**) Quantitative analysis of AIM2 mean fluorescence intensity. *n* = 3, ** *p* < 0.01, compared with CG or sham group. Statistical analysis was performed using one-way ANOVA followed by Tukey’s post hoc test for comparisons among three groups. Data are expressed as means ± standard error of mean (SEM).

As shown in Figure 6, immunofluorescence staining was performed to further evaluate AIM2 expression in the CeA at 1 d. Positive fluorescence was observed in all three groups, with a visibly stronger signal in the experimental group compared to the other two groups. A quantitative analysis of mean fluorescence intensity showed a significant difference among the groups (one-way ANOVA, *F*(2, 6) = 17.63, *p* = 0.0031, *n* = 3 per group). Tukey’s post hoc test showed that AIM2 fluorescence intensity was significantly increased in the experimental group compared with the control group (CG vs. EG, adjusted *p* = 0.0038) and the sham group (sham vs. EG, adjusted *p* = 0.0075). No significant difference was observed between the control and sham groups. These results further confirmed the upregulation of AIM2 expression in the CeA after experimental tooth movement.

### 2.7. Protein Expression of AIM2 in CeA After ETM at Different Time Points

**Figure 7:**
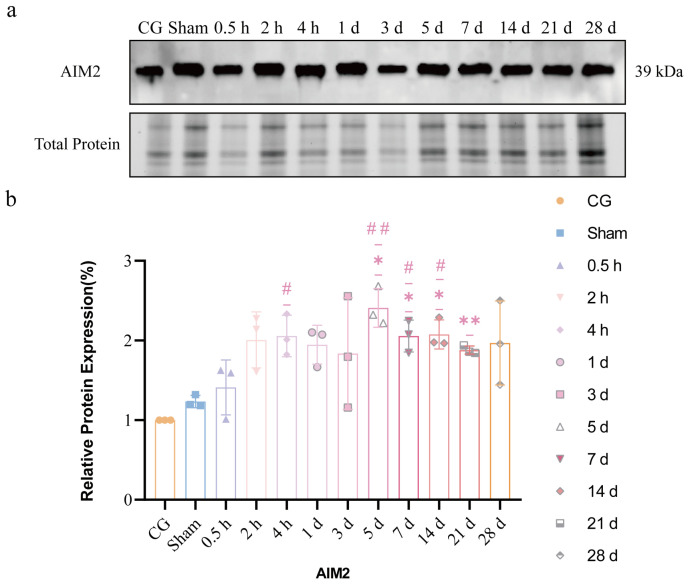
Western blots of AIM2 in CeA at different time points. (**a**) AIM2 expressed in CeA. (**b**) Quantitative analysis. *n* = 3, * *p* < 0.05, ** *p* < 0.01, compared with CG. # *p* < 0.05, ## *p* < 0.01, compared with sham group. Statistical analysis was performed using one-way ANOVA followed by Tukey’s post hoc test for comparisons among three groups. Data are expressed as means ± standard error of mean (SEM).

As shown in Figure 7, compared with the CG, both the sham group and the EG exhibited varying degrees of increased AIM2 expression. To evaluate the incremental effect of force application, an ordinary one-way ANOVA was performed, confirming a significant overall difference in AIM2 expression among the groups (*F*(11, 24) = 4.565, *p* = 0.0009, *n* = 3 per group). Post hoc analysis using Tukey’s post hoc test revealed that compared to the sham group, AIM2 expression in the experimental group remained significantly elevated at 4 h (*p* = 0.0387), 7 d (*p* = 0.0388), and 14 d (*p* = 0.0327) and peaked at 5 d (*p* = 0.0017).

### 2.8. Protein Expression of AIM2 in CeA After ETM at Pain and Anxiety Peak Time Points

**Figure 8:**
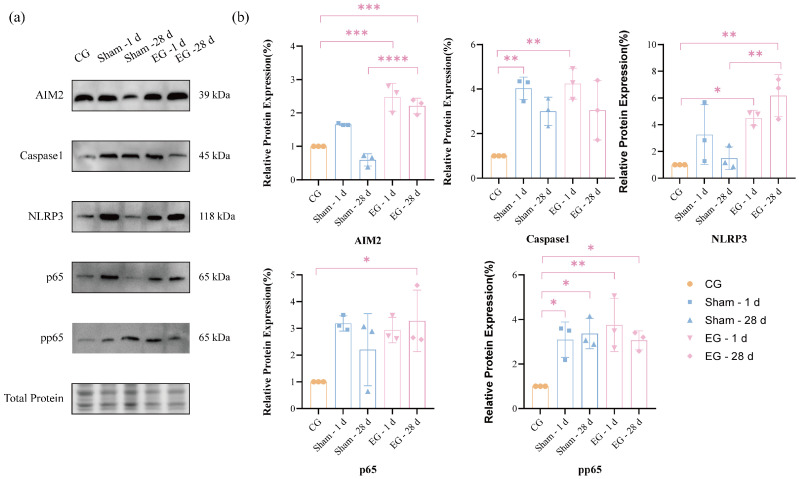
Western blots of AIM2 in CeA at 1 d and 28 d. (**a**) Relative expression in CeA. (**b**) Quantitative analysis. *n* = 3, * *p* < 0.05, ** *p* < 0.01, *** *p* < 0.001, **** *p* < 0.0001, compared with sham group. Statistical analysis was performed using one-way ANOVA followed by Tukey’s post hoc test for comparisons among three groups. Data are expressed as means ± standard error of mean (SEM).

As shown in Figure 8, the Western blot results showed that AIM2 expression levels in the EG were significantly higher than those in the other two groups (one-way ANOVA, *n* = 3 per group). Caspase-1 expression levels in both the EG and sham group were markedly higher than those in the CG (*F*(4, 10) = 8.452, *p* = 0.003). NLRP3 expression levels in the EG were higher than those in the other two groups and gradually increased over time (*F*(4, 10) = 8.084, *p* = 0.0035). pp65 expression levels were significantly higher in both the sham group and EG compared to the CG (*F*(4, 10) = 6.380, *p* = 0.0081).

### 2.9. Cannula Administration Influencing AIM2 Regulates Tooth Movement Pain- and Anxiety-like Behavior

**Figure 9:**
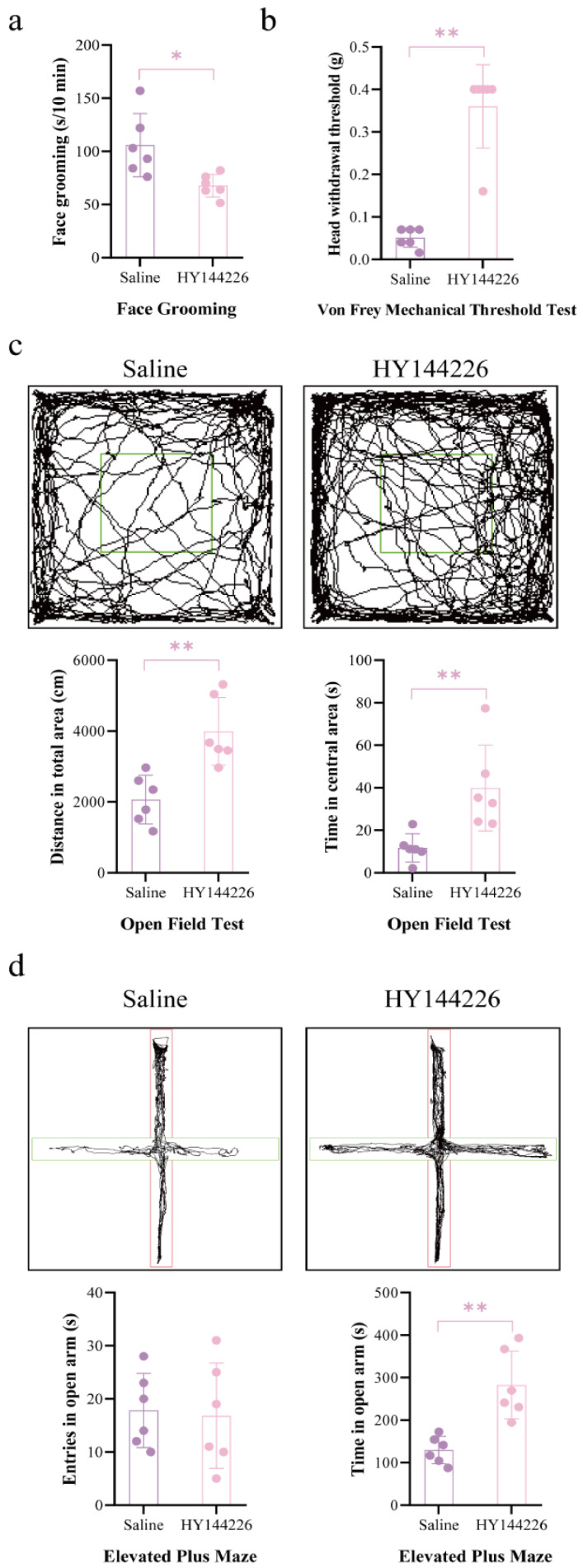
Effects of CeA cannula administration of HY144226 on tooth movement-induced pain- and anxiety-like behaviors. (**a**) Face grooming behavior after drug administration. (**b**) Von Frey mechanical threshold test. (**c**) Open field test results, including total distance in open field and time spent in central area. (**d**) Elevated plus maze results, including entries into open arms and time spent in open arms. Red: closed arms; Green: open arms. *n* = 6, * *p* < 0.05, ** *p* < 0.01, compared with saline group. Statistical analysis was performed using unpaired two-tailed *t* test. Data are expressed as means ± standard error of mean (SEM).

As shown in Figure 9, the intra-CeA administration of HY144226 significantly affected tooth movement-induced pain- and anxiety-like behaviors. Compared with the saline group, mice receiving HY144226 showed a significant reduction in face grooming behavior after drug administration (unpaired *t* test, t = 2.951, df = 10, *p* = 0.0145, *n* = 6 per group).

In the von Frey mechanical threshold test, HY144226 significantly increased the head withdrawal threshold compared with saline treatment (unpaired *t* test, t = 7.528, df = 10, *p* < 0.0001, *n* = 6 per group). These results suggest that the CeA administration of HY144226 alleviated tooth movement-induced pain-related behaviors.

In the open field test, HY144226-treated mice exhibited significantly increased locomotor distance compared with saline-treated mice (unpaired *t* test, t = 4.012, df = 10, *p* = 0.0025, *n* = 6 per group). The mean total distance increased from 2066 cm in the saline group to 3991 cm in the HY144226 group. In addition, the time spent in the central area was significantly increased after HY144226 administration (unpaired *t* test, t = 3.237, df = 10, *p* = 0.0089, *n* = 6 per group).

In the elevated plus maze test, HY144226 significantly increased the time spent in the open arms compared with the saline group (unpaired *t* test, t = 4.362, df = 10, *p* = 0.0014, *n* = 6 per group). However, no significant difference was observed in the number of open arm entries between the two groups (unpaired *t* test, t = 0.2017, df = 10, *p* = 0.8442, *n* = 6 per group). Together, these findings indicate that the CeA administration of HY144226 reduced tooth movement-induced pain-related behaviors and partially alleviated anxiety-like behaviors.

## 3. Discussion

Pain is one of the most common adverse reactions during orthodontic treatment, causing not only physical discomfort but also negative emotions such as anxiety and tension [13]. This study established an ETM model in mice to evaluate changes in pain- and anxiety-like behaviors following orthodontic force application. The results showed that mice exhibited pronounced pain behaviors shortly after force application. Both face grooming and von Frey test scores began rising at 0.5 h, peaked at 1 d, then gradually declined, returning to baseline levels by 7 d. These findings are consistent with previous research on orthodontic pain [4,5,6,14]. These findings suggest that the model effectively reflects the onset and resolution of early orthodontic pain. Anxiety-like behaviors were observed beginning approximately 14 d post-force application and appeared to peak around 28 d. Given the temporal dissociation between mechanical hypersensitivity and later behavioral changes, these findings suggest that sustained peripheral stimulation leads to subsequent behavioral alterations. This process potentially involves central nervous system mechanisms. Interestingly, our molecular study revealed that compared to the untreated control group, the expression levels of specific central signaling proteins were partially elevated in the sham group. This suggests that the sham surgery procedure itself may influence central signaling. The presence of the intraoral device did not cause significant pain response, but it still induced a certain degree of discomfort. Thus, it is important to note that the upregulation of these proteins was significantly greater in the ETM group compared to the sham group. This significant difference confirms that, although oral instrumentation does indeed produce behavior stimulation, the application of active orthodontic forces is the primary factor triggering a pronounced central nervous system inflammatory response and subsequent emotional and behavioral changes. In summary, the tooth movement model established in this study reflects the patterns of pain- and anxiety-like behaviors during orthodontic treatment. It provides a reliable experimental foundation for exploring the underlying central regulatory mechanisms.

The amygdala serves as a critical emotional regulation center, participating in the processing of various emotional responses, particularly playing a vital role in pain, anxiety, and fear reactions [15]. Recent studies indicate that the CeA plays a significant role in the generation of chronic pain and anxiety behaviors. Through interactions with regions such as the thalamus and cortex, the CeA regulates emotional expression and pain perception. AIM2 initiates inflammasome formation, thereby promoting the release of inflammatory mediators [16]. This function of AIM2 has been validated in numerous disease models. AIM2 inflammasomes regulate neuronal morphology and influence anxiety and memory in mice [17]. In orthodontic tooth movement models, pain responses induced by force application activate the CeA-AIM2 pathway, thereby driving central inflammatory responses. This discovery provides new insights into the role of the CeA-AIM2 pathway in oral pain and emotional regulation. Furthermore, it offers a potential research direction for targeting this pathway to mitigate adverse reactions during orthodontic treatment. The results showed that after establishing the tooth movement model, AIM2 expression increased to varying degrees at different time points, preliminarily confirming that changes in AIM2 expression may be associated with tooth movement pain and anxiety.

Research indicates that the mechanism of action for AIM2 may be associated with the NLRP3-NF-κB signaling pathway, with caspase-1 potentially playing a crucial role as a key downstream molecule [18]. Therefore, this study examined the expression of proteins related to this signaling pathway at the time points when behavioral manifestations were the most pronounced: pain (1 d) and anxiety (28 d). The results indicated that both p65 and pp65 levels were significantly elevated in the CeA. NLRP3 expression increased with prolonged exposure time, suggesting that NLRP3 plays a crucial role in anxiety-like behavior in mice. Collectively, this study reveals the role of AIM2 within the CeA in tooth movement-induced pain. These findings offer deeper insights into the emotional and sensory aspects of orthodontic treatment. Furthermore, by identifying potential molecular targets, this research provides a foundation for future medications to alleviate orthodontic pain and anxiety.

The behavioral results indicate the crucial role of AIM2 within the CeA in tooth movement-induced pain and provides potential targets for clinical intervention, but there still remain limitations. First, in the OFT and EPM, reduced center time and open arm exploration are commonly interpreted as anxiety-like behaviors. In our study, nociceptive behaviors peaked within 1 d and resolved by 7 d, whereas anxiety-like behaviors emerged after 14 d and persisted until 28 d. This temporal dissociation suggests that the affective alterations are not solely attributable to acute pain-induced locomotor suppression. Among the various measurements for assessing anxiety levels, these locomotor-related parameters remain controversial. Previous studies have adopted different approaches. For example, anxiety-like behavior was primarily assessed using open arm time in the OFT and center time in the EPM [19], without other detailed analysis. In another study, both time-based indices and locomotor-related measures were evaluated, but total distance did not show a significant trend, which is consistent with our findings [20]. These observations suggest that time-based measures remain widely used indicators of anxiety-like behavior in chronic pain research. Nonetheless, we acknowledge that incorporating additional behavioral paradigms or locomotion-independent assays in future studies would further strengthen the interpretation. Second, we prepared three independent samples for the Western blot analysis. Although our findings show some similarity to previous reports [21,22], it is still necessary to expand the sample size. Increasing the number of samples would further strengthen the reliability and generalizability of the results. Last, this study predominantly employed mouse models. Although these models effectively simulate nociceptive responses associated with orthodontic treatment, the extent to which they fully replicate human emotional experiences and pain mechanisms during actual clinical procedures remains to be further validated. Future investigations should therefore focus on translating this methodology into clinically applicable approaches.

## 4. Materials and Methods

### 4.1. Experimental Design and Animals

**Figure 10:**
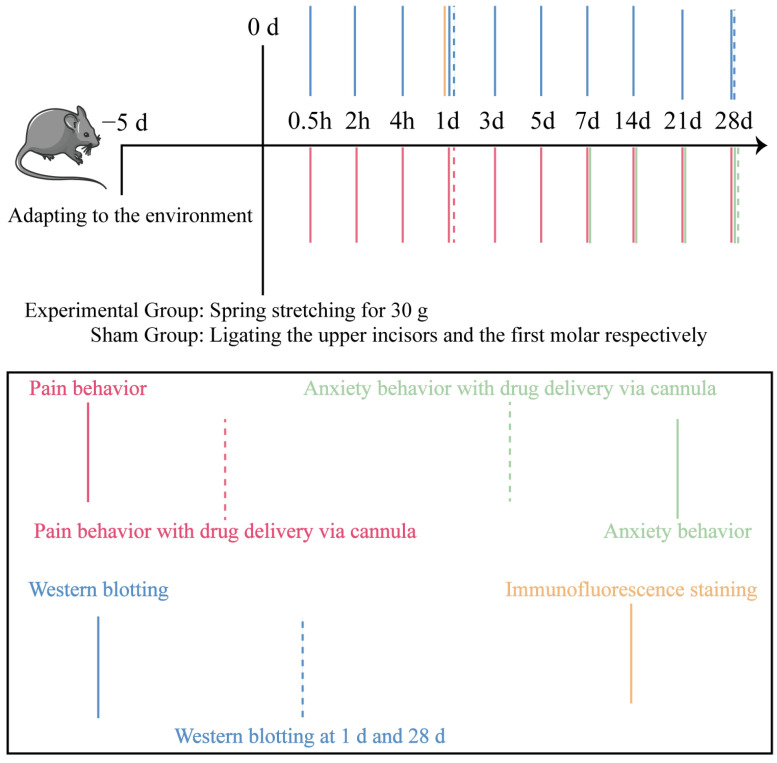
Behavioral and molecular biology experimental workflow diagram.

This study used 8-week male C57BL/6J mice weighing approximately 20–25 g, supplied by the Experimental Animal Center of Xi’an Jiaotong University Medical Center (Xi’an, China). A total of 81 mice were used in this study. Among them, 72 mice were used for the initial behavioral assessments and Western blot experiments, including 12 experimental cohorts with 6 mice in each cohort. Additional mice were used for the newly added immunofluorescence experiment, with 3 mice in each group. The mice were assigned to a control group, sham group, or experimental group according to the corresponding experimental endpoint. Mice were housed in a thermostatically controlled environment (21 ± 1.5 °C) under a 12 h light/dark cycle (lights off at 8:00 p.m.). Mice were randomly divided into three groups using a computer-generated randomization sequence, control group (CG), sham group, and experimental group (EG), and each group had 6 mice. To minimize the confounding effects of consecutive testing, behavioral assays were conducted in a strictly controlled sequence from the least to the most stressful, and all testing and subsequent data analyses were performed by investigators blinded to the group allocations. Micro-CT was employed to verify model validity. The experimental design is shown in Figure 10. In accordance with ethical guidelines for animal research, every effort was made to minimize animal discomfort. The experimental procedures were conducted in accordance with the National Institutes of Health Guidelines for the Care and Use of Laboratory Animals and were approved by the Institutional Animal Care and Use Committee of Xi’an Jiaotong University (Approval NO. 2020-386).

### 4.2. Experimental Tooth Movement (ETM)

Mice were anesthetized with Avertin (2.5%, 10 μL/g, intraperitoneal injection). During the procedure and subsequent recovery, body temperature was maintained at 37 °C using a thermostatic heating pad. A custom-made stainless steel spring ligature was applied between the maxillary right incisor and maxillary right first molar to apply traction force, pulling the maxillary right first molar mesially [23]. A dynamometer measured the tension value of the stretched spring to ensure consistent results across all models. After ligation, glass ionomer cement was used to secure the spring and prevent dislodgement. For mice in the sham group, the same anesthesia, oral manipulation, and ligation procedures were performed as in the experimental group, but the orthodontic spring was not installed to apply mechanical force. Therefore, no active orthodontic force was delivered in the sham group, and the force level was considered 0 g. Additional trauma to the animals was minimized during the procedure. Postoperatively, mice in all groups were provided with a softened diet placed on the cage floor to ensure adequate nutritional intake. Animal health, including body weight and daily food consumption, was closely monitored. Pre-defined exclusion criteria were strictly enforced: animals exhibiting severe distress, appliance detachment, visible oral mucosal ulcerations, or a body weight loss exceeding 15% of their baseline weight were immediately excluded from this study.

### 4.3. Micro-CT Performance Evaluation

To validate the orthodontic tooth movement model, maxillary bone samples were collected from mice two weeks after modeling and subjected to high-resolution scanning using micro-CT. Tooth movement distance was assessed by measuring the shortest distance between the right maxillary first and second molars. Successful ETM modeling was determined when significant mesial movement of the first molar was observed, accompanied by typical pathological changes such as alveolar bone resorption on the pressure side.

### 4.4. Face Grooming Test

All behavioral tests were performed during the same time period (6:00–10:00 p.m.). This testing period coincided with the animals’ active (dark) phase, as they were maintained on a standard 12/12 h light/dark cycle (lights off at 8:00 p.m.). Face grooming tests were conducted in a quiet, independent laboratory with constant background noise (approximately 45 dB). Prior to the experiment, mice were placed in transparent square plastic cages (40 × 40 × 40 cm) for 30 min of free activity to fully acclimate to the environment and prevent stress-related behaviors. Subsequently, a video recording system captured 10 min of facial self-grooming behaviors, such as the paw-wiping of the mouth corners. Laboratory temperature and lighting conditions were maintained constant, and test cages were cleaned with 70% ethanol after each session to eliminate residual odors. Specifically, all behavioral assessments were conducted under dim red light illumination to prevent circadian disruption. Analysis was performed by two experimenters blinded to treatment outcomes, with the average of three trials serving as the estimated test result for each mouse.

### 4.5. Von Frey Test

To minimize stress and visual interference, mice were placed in individual opaque chambers and habituated to the environment for 30 min daily for three consecutive days prior to testing. Mechanical stimulation was applied to mice using a series of von Frey filaments with varying force values. The test site was the skin at the right corner of the mouth. Stimulation began with fibers of medium intensity (initial force of 0.04 g) and was adjusted according to the mouse’s behavioral response using the up–down paradigm [24]. Defensive reactions such as scratching the mouth corner or rapid head withdrawal can be regarded as an active response. The 50% mechanical withdrawal threshold was calculated and served as the mechanical pain threshold for each mouse. Measurements were repeated three times per animal, with subsequent statistical analysis conducted by two experimenters blinded to the experimental conditions.

### 4.6. Open Field Test (OFT)

The experiment was conducted in a transparent square plastic box (40 × 40 × 40 cm), with the bottom divided into 25 equal-sized squares [25]. The central 9 squares were defined as the central zone. Mice were gently placed in the center zone to freely explore for 10 min, with the entire process recorded by an overhead camera system. Then the number of entries into the center zone and the duration of stay within it were analyzed, thereby assessing anxiety-like behavior. Two independent observers, unaware of the treatment, then analyzed the test results.

### 4.7. Elevated Plus Maze (EPM)

The experimental apparatus consists of two open arms (30 × 5 × 0.5 cm) and two closed arms (30 × 5 × 15 cm), with the arm junctions forming a cross-shaped structure. The apparatus stands 50 cm tall. Mice were placed at the center point with their heads facing the open arms and allowed to freely explore for 10 min, with the entire process recorded by an overhead camera system. The number of entries into the open arms and the duration of stay in the open arms were analyzed [26]. Subsequently, two independent observers, blinded to the treatment, analyzed the test results.

### 4.8. Immunofluorescence Staining

Frozen brain sections were collected at 1 day after experimental tooth movement and were first permeabilized and blocked in a Tris-buffered saline (TBS) solution containing 10% goat serum (Sigma-Aldrich, St. Louis, MO, USA; G9023) and 0.3% Triton X-100 for 60 min at room temperature. The slices were subsequently incubated with a primary antibody against AIM2 (Santa Cruz Biotechnology, Dallas, TX, USA; #sc-293174, 1:100) at 4 °C overnight. On the following day, sections were washed and treated with corresponding fluorophore-conjugated secondary antibodies at room temperature for 2 h. Finally, DAPI (Southern Biotech, Birmingham, AL, USA; 0100-20) was applied for 5 min to counterstain the nuclei, and the sections were mounted for fluorescence microscopy imaging.

### 4.9. Western Blotting Analysis

After harvesting mouse brain CeA regions, tissues were lysed using RIPA buffer. Samples were prepared on 10% TGX non-staining polyacrylamide gel (Bio-Rad, Hercules, CA, USA). Total protein data were acquired using the GelDoc Go imaging system (Bio-Rad, Hercules, CA, USA) and subsequently transferred to PVDF membranes. After blocking with 5% non-fat dry milk at room temperature for 1 h, the primary antibody was incubated overnight at 4 °C. The following day, the membrane was washed three times with TBST for 10 min each, followed by incubation with the secondary antibody. The primary antibodies used were anti-AIM2 (Cell Signaling Technology, Danvers, MA, USA; #6360, 1:1000), anti-caspase-1 (CST, #24232, 1:1000), anti-NLRP3 (CST, #15101, 1:1000), anti-NF-κB p65 (CST, #8242, 1:1000), and anti-phospho-NF-κB p65 (Ser536) (CST, #3033, 1:1000). The secondary antibody was HRP-linked anti-rabbit IgG (CST, #7074, 1:1000). Band optical densities were quantified using ImageJ software (Version 1.54p) [27]. To ensure the reliability of the results, all experiments were performed using three independent biological replicates (*n* = 3 per group), with technical repeats conducted to confirm procedural consistency.

### 4.10. Drug Delivery via Cannula

Mice were fixed in a stereotaxic instrument. After a midline scalp incision, the skull surface was leveled using bregma and lambda as reference points. With bregma serving as the zero-coordinate origin, bilateral guide cannulae were implanted into the central nucleus of the amygdala (CeA; AP: −1.0 mm, ML: ±2.8 mm, DV: −3.8 mm) and anchored with glass ionomer cement (Shangchi, Changshu, China). Before behavioral assays, NLRP3/AIM2-IN-3 (MedChemExpress, Monmouth Junction, NJ, USA) was bilaterally infused via an internal cannula (0.2 mM, 300 nL/side). The internal cannula remained in position for 3 min post-infusion to ensure complete diffusion.

### 4.11. Statistical Analysis

All experimental data were analyzed using GraphPad Prism 8.0 (GraphPad Software, San Diego, CA, USA). A one-way ANOVA combined with Tukey’s post hoc test was used to evaluate significant differences among three or more groups; a two-way ANOVA was applied to examine significant differences between different groups within the same treatment. A two-way repeated measures ANOVA was used for comparing data from multiple factors with repeated measurements. Data are expressed as means ± standard error of the mean (SEM). *p* < 0.05 indicates statistically significant differences.

## 5. Conclusions

This study suggests that experimental tooth movement is associated with specific molecular responses in the CeA. The upregulation of AIM2 within the CeA following force application in mice was observed, accompanied by the increased expression of signaling components, including NLRP3, caspase-1, p65 and pp65. These findings indicate that the AIM2/NLRP3/NF-κB pathways may play a potential role in the central processing of orthodontic pain and related affective responses. Our results provide new insights into the possible molecular mechanisms underlying orthodontic tooth movement.

## Data Availability

The data that support the findings of this study are available on request from the corresponding author.

## Abbreviations

The following abbreviations are used in this manuscript:

AIM2	Absent in melanoma 2
CeA	Central nucleus of the amygdala
CG	Control group
EG	Experimental group
EPM	Elevated plus maze
ETM	Experimental tooth movement
NLRP3	NOD-like receptor family pyrin domain-containing 3
OFT	Open field test

## References

[1] Cenzato N., Nobili A., Maspero C. (2021). Prevalence of Dental Malocclusions in Different Geographical Areas: Scoping Review. Dent. J..

[2] Long H., Wang Y., Jian F., Liao L.N., Yang X., Lai W.L. (2016). Current advances in orthodontic pain. Int. J. Oral Sci..

[3] Wang J., Tang X., Shen Y., Shang G., Fang L., Wang R., Xu Y. (2015). The correlations between health-related quality of life changes and pain and anxiety in orthodontic patients in the initial stage of treatment. Biomed. Res. Int..

[4] Qiao H., Gao Y., Huang Q., Jia R. (2020). The central nucleus of the amygdala lesion attenuates orthodontic pain during experimental tooth movement in rats. Brain Behav..

[5] Wang R., Wang W., Kang Y., Jiang Y., Tang X., Shu Y., Zhou J., Hu Z., Wang S., Qiao H. (2025). Increased Expression of TRPV1 in the Central Nucleus of the Amygdala Is Involved in Orthodontic Pain in Rats. Int. J. Mol. Sci..

[6] Wang R., Fang D., Kang Y., Wang W., Guo Y., Kang H., Fan J., Yin S., Deng Y., Hu Z. (2026). Rising TRPV4 expression in the central nucleus of amygdala is involved in tooth movement pain. Physiol. Behav..

[7] Lin Y.L., Yang Z.S., Wong W.Y., Lin S.C., Wang S.J., Chen S.P., Cheng J.K., Lu H., Lien C.C. (2022). Cellular mechanisms underlying central sensitization in a mouse model of chronic muscle pain. Elife.

[8] Wang L., Sun L., Byrd K.M., Ko C.C., Zhao Z., Fang J. (2020). AIM2 Inflammasome’s First Decade of Discovery: Focus on Oral Diseases. Front. Immunol..

[9] Oh S., Lee J., Oh J., Yu G., Ryu H., Kim D., Lee S. (2023). Integrated NLRP3, AIM2, NLRC4, Pyrin inflammasome activation and assembly drive PANoptosis. Cell. Mol. Immunol..

[10] Sharma B.R., Karki R., Kanneganti T.D. (2019). Role of AIM2 inflammasome in inflammatory diseases, cancer and infection. Eur. J. Immunol..

[11] Chen Y., Ye X., Escames G., Lei W., Zhang X., Li M., Jing T., Yao Y., Qiu Z., Wang Z. (2023). The NLRP3 inflammasome: Contributions to inflammation-related diseases. Cell. Mol. Biol. Lett..

[12] Peng L., Wen L., Shi Q.F., Gao F., Huang B., Meng J., Hu C.P., Wang C.M. (2020). Scutellarin ameliorates pulmonary fibrosis through inhibiting NF-κB/NLRP3-mediated epithelial-mesenchymal transition and inflammation. Cell Death Dis..

[13] Xie Y., Zheng X., Li Y., He J., Wang P., Han X. (2024). The effect of somatic pain and comorbid mental distress on oral health-related quality of life in orthodontic patients. Clin. Oral Investig..

[14] Gao Y., Wang R., Liu Q., Zhou B., Qiao H. (2024). Effect of acetaminophen on relieving orthodontic pain with clear aligner based on GAD-7: A retrospective research. Heliyon.

[15] Cai Y.Q., Wang W., Paulucci-Holthauzen A., Pan Z.Z. (2018). Brain Circuits Mediating Opposing Effects on Emotion and Pain. J. Neurosci..

[16] Rossi C., Salvati A., Distaso M., Campani D., Raggi F., Biancalana E., Tricò D., Brunetto M.R., Solini A. (2022). The P2X7R-NLRP3 and AIM2 Inflammasome Platforms Mark the Complexity/Severity of Viral or Metabolic Liver Damage. Int. J. Mol. Sci..

[17] Wu P.J., Liu H.Y., Huang T.N., Hsueh Y.P. (2016). AIM 2 inflammasomes regulate neuronal morphology and influence anxiety and memory in mice. Sci. Rep..

[18] Huo H., Wu H., Ma F., Li X., Liao J., Hu L., Han Q., Li Y., Pan J., Zhang H. (2022). N-acetyl-L-cysteine ameliorates hepatocyte pyroptosis of dog type 1 diabetes mellitus via suppression of NLRP3/NF-κB pathway. Life Sci..

[19] Song Q., Wei A., Xu H., Gu Y., Jiang Y., Dong N., Zheng C., Wang Q., Gao M., Sun S. (2024). An ACC-VTA-ACC positive-feedback loop mediates the persistence of neuropathic pain and emotional consequences. Nat. Neurosci..

[20] He M., Chen Y.X., Feng P.P., Chen J., Xu C., Zhou S.T., Liu B.Y., He X.F., Shao X.M., Fang J.Q. (2024). Berberine alleviates chronic pain-induced anxiety-like behaviors by inhibiting the activation of VLT-projecting cACC (Cg2) neurons. Commun. Biol..

[21] Anderson G.J., Cipolla C., Kennedy R.T. (2011). Western blotting using capillary electrophoresis. Anal. Chem..

[22] Zhang Y., Naguro I., Herr A.E. (2019). In Situ Single-Cell Western Blot on Adherent Cell Culture. Angew. Chem. Int. Ed. Engl..

[23] Klein Y., David E., Pinto N., Khoury Y., Barenholz Y., Chaushu S. (2024). Breaking a dogma: Orthodontic tooth movement alters systemic immunity. Prog. Orthod..

[24] Han Y., Ai L., Song L., Zhou Y., Chen D., Sha S., Ji R., Li Q., Bu Q., Pan X. (2024). Midbrain glutamatergic circuit mechanism of resilience to socially transferred allodynia in male mice. Nat. Commun..

[25] Parrella E., Bellucci A., Porrini V., Benarese M., Lanzillotta A., Faustini G., Longhena F., Abate G., Uberti D., Pizzi M. (2019). NF-κB/c-Rel deficiency causes Parkinson’s disease-like prodromal symptoms and progressive pathology in mice. Transl. Neurodegener..

[26] Zelko M.D., Robinson S.R., Hill-Yardin E.L., Nasser H. (2025). Resolving anxiety-like behaviour inconsistencies in the elevated plus maze by tracking exploration depth and timing. Behav. Res. Methods.

[27] Xu S.Y., Bian H.J., Shu S., Xia S.N., Gu Y., Zhang M.J., Xu Y., Cao X. (2021). AIM2 deletion enhances blood-brain barrier integrity in experimental ischemic stroke. CNS Neurosci. Ther..

